# *In vitro* and *in vivo* apatinib inhibits vasculogenic mimicry in melanoma MUM-2B cells

**DOI:** 10.1371/journal.pone.0200845

**Published:** 2018-07-27

**Authors:** Zong-Jun-Lin Liu, Yu-Juan Zhou, Rui-Lin Ding, Fang Xie, Shao-Zhi Fu, Jing-Bo Wu, Ling-Lin Yang, Qing-Lian Wen

**Affiliations:** 1 Department of Oncology, Affiliated Hospital of Southwest Medical University, Luzhou, Sichuan, China; 2 Institute of Drug Clinical Trial/GCP Center, Affiliated Hospital of Southwest Medical University, Luzhou, Sichuan, China; University of South Alabama Mitchell Cancer Institute, UNITED STATES

## Abstract

The effect of apatinib on the formation of vasculogenic mimicry (VM) was studied in a malignant melanoma cell line. MUM-2B cells cultured in three-dimensional Matrigel were treated with varying concentrations (0, 0.01, 0.05, 0.1, 0.5 μmol/L) of apatinib to test its effect on VM in vitro, followed by MTT proliferation and transwell invasion assays to determine the effect of apatinib on cell proliferation and invasion of MUM-2B cells. In vivo, we used a melanoma cancer model to test the effect of short-term apatinib (100, 200, 300 mg/kg) treatment on VM. Western blotting, immunohistochemistry staining, and CD31-PAS dual staining were performed to assess the expression of VEGFR-2, ERK-1/2, PI3K, and MMP-2, and formation of VM. The results showed apatinib-treated groups formed a lesser number of VM in 3D matrigel, while the cell viability in MTT proliferation assay and the number of migration cells in transwell invasion assay were significantly lower in apatinib-treated groups. In addition, short-term apatinib treatment inhibited angiogenesis, VM formation, and tumor growth in models of melanoma cancer. Mice in apatinib-treated groups showed a markedly reduced expression of VEGFR-2, ERK-1/2, PI3K, and MMP-2. In summary, apatinib could inhibit the expression of VEGFR-2, and downregulate the ERK1/2/PI3K/MMP-2 signaling cascade, which may be one of the underlying mechanisms by which apatinib inhibits angiogenesis and the development of VM in models of melanoma cancer, and restrains the formation of VM by MUM-2B cells. Apatinib shows inhibitory effects on cell proliferation and invasion of MUM-2B cells, which is a close relationship with the VM.

## Introduction

Anti-angiogenic therapy is one of the most promising methods in the treatment of cancer. However, a number of limitations are observed in current antiangiogenic therapies[1]. Single-agent use of antiangiogenesis appears to be insufficient to improve patient survival[2]. This is in part because tumor vasculature is more complex than expected, and alternative mechanisms for re-vascularization might be taking place. The angiogenesis inhibitor may cause hypoxia in tumor cells, which promotes the formation of VM to provide blood supply for tumor cells[3,4].

Vasculogenic mimicry (VM), a new model of tumor microcirculation found in melanoma in the last 10 years, is a vascular channel-like structure composed of tumor cells, but lack of endothelial cells, which shows positive staining for periodic acid-Schiff (PAS) and negative staining for CD31. VM could provide highly aggressive malignant tumor cells with adequate blood supply. The presence of VM has a close relationship with the occurrence, development, metastasis, and long-term adverse prognosis of the tumor[5–7]. VM is independent of endothelial cells, which may partly explain why angiogenesis inhibitors have not achieved the expected success. Previous studies have shown that bevacizumab could promote the formation of VM[3], while endostatin had no obvious inhibitory effect on the formation of VM in human melanoma cells[8]. Therefore, identifying molecules that suppress VM formation may provide targets for cancer therapy.

Although the mechanism of VM is not yet clear, studies have found that the ERK-1/PI3K/MMP-2 signaling cascade might be critical for VM formation[9]. In addition, vascular endothelial growth factor receptor-2 (VEGFR-2), like most receptor tyrosine kinases (RTKs), induced proliferation via activation of the classical extracellular signal-regulated kinases (ERK) pathway. Therefore, the VEGFR-2 on the surface of tumor cells may be associated with the formation of VM[10,11].

Apatinib, also known as YN968D1, is a new agent for anti-angiogenic therapy, which was also confirmed to be a safe and effective small molecule anti-angiogenic targeted drug for advanced gastric cancer, with the independent intellectual property rights of China in 2014. Giandomenico et al. have shown that the mechanism of apatinib is mediated by its binding to the intracellular ATP-binding site of VEGFR-2, thus blocking its phosphorylation and restraining its downstream proangiogenic signaling pathways, similar to most receptor tyrosine kinases (RTKs)[12–14]. We believe that apatinib could both restrain angiogenesis and inhibit the formation of VM.

We have explored the effect of apatinib on the formation of VM and detected its possible related mechanism in a human malignant melanoma cell line (MUM-2B).

## Materials and methods

### Cell culture

Human invasive choroidal melanoma cells (MUM-2B) were purchased from the GuangZhou Jennio Biotech Co., Ltd. (China). MUM-2B cells were grown in Roswell Park Memorial Institute-1640 medium (RPMI-1640, HyClone, Thermo Scientific, USA). The media were both supplemented with 10% fetal bovine serum (HyClone, Thermo Scientific), 100 IU/mL of penicillin G sodium, and 100 mg/mL of streptomycin sulfate. The cells were both maintained at 37°C in an incubator with 95% air and 5% CO_2_ in a humidified atmosphere.

### Animal models

Sixty female BALB/c mice (4–5 weeks of age) were purchased from the Laboratory Animal Center of the Chongqing Municipality (China). Mice were kept in a specific-pathogen-free (SPF) laminar air flowbox and were fed with sterile food pellets and water *ad libitum*. The subcutaneous melanoma cancer model was established by injecting 100 mL suspension of MUM-2B cells (1×10^7^ cells/mL) into the right armpit of BALB/c mice. The cells were allowed to grow for 2 weeks until the tumors were approximately 200 mm^3^ in volume. According to clinical doses used *in vivo* from a previous study, sixty tumor bearing mice were assigned into four groups (n = 15): NS group, 100 mg/kg Apatinib group, 200 mg/kg Apatinib group, 300 mg/kg Apatinib group (Jiangsu Hengrui Medicine Co., Ltd, China), that received treatments administered orally for 2 weeks. Mice were sacrificed by cervical dislocation on day 14, and the tumor tissues and blood samples were collected for further analysis.

During the treatment, tumor size (length and width) was measured using calipers every days. Tumor volumes were calculated with the formula $\text{V}=\frac{a\times b^{2}}{2}$, where V is the tumor volume, a is the longest axis and b is the perpendicular shorter tumor axis. A tumor growth curve was plotted based on tumor size and length of survival, in days, after treatment. The tumor volume inhibition rate on day 14 was calculated according to the following equation [15].

$$\text{Inhibition}\;\text{rate}\;\%=\left(1-\frac{\text{Volume}\;\text{Day}\;1\;\text{treatment}\;\text{group}-\text{Volume}\;\text{Day}\;14\;\text{treatment}\;\text{group}}{\text{Volume}\;\text{Day}\;1\;\text{control}\;\text{group}-\text{Volume}\;\text{Day}\;14\;\text{control}\;\text{group}}\right)\times 100\%$$

In order to alleviate suffering of experiment animals, we optimized the feeding environment and diet, moreover we used the cervical dislocation to execute animals. All animal care and experimental procedures were approved and performed according to the Institutional Animal Care and Use Guidelines. Animal experiments were approved by the Institutional Animal Care and Treatment Committee of Southwest Medical University (China).

### Three-dimensional cell culture

Three-dimensional Matrigel was produced as follows: 300 μl of Matrigel (Becton, Dickinson and Company, USA) was dropped onto a flat bottom 24-well tissue culture plate and polymerized for 1 h at room temperature, followed by 30 min of incubation at 37°C in a humidified 5% CO_2_ incubator. MUM-2B cells (1×10^5^) were seeded into the three-dimensional Matrigel. RPMI-1640 medium (RPMI-1640; HyClone, Thermo Scientific, USA) supplemented with 10% FBS (HyClone, Thermo Scientific, USA) was changed every 24 h, stopped after 7 days[8]. Then, MUM-2B cells cultured in the three-dimensional culture Matrigel were treated with varying concentrations (0, 0.01, 0.05, 0.1, 0.5 μmol/L) of apatinib (Jiangsu Hengrui Medicine Co., Ltd, China) according to clinical doses used *in vitro* from a previous study[16]. Apatinib supplemented with RPMI-1640 medium and 10% FBS was changed every 24 h in the three-dimensional culture Matrigel, was discontinued after 7 days.

The cell morphology and vascular structures were observed under an inverted phase contrast microscope. Ten fields of vision of the phase contrast microscope of each pathological section were selected to count the number of VM at 200× magnification, and to calculate the average number of VM that are quantification of the vasculogenic mimicry density (VMD) in the three-dimensional culture Matrigel, as reported previously. All these counts were blindly performed.

### Proliferation assay

For the proliferation assay, the MUM-2B cell suspensions (5 ×10^4^) were seeded onto a flat bottom 96-well plate and incubated at 37 °C for 48h. These cells were treated with different concentrations of Apatinib (0, 0.01, 0.05, 0.1, 0.5 μmol/L) for different times. Cell proliferation activity was determined by the MTT (3-[4,5-di-methylthiazol-2-yl]-2,5 diphenyltetrazolium bromide) assay. MTT was purchased from Beijing Iptonic Technology Co. Ltd (China).

$$\text{Cell}\;\text{viability}=1-\frac{OD_{treatment}-OD_{M}}{OD_{control}-OD_{M}}\times 100\%$$

### Invasion assay

The invasion assay was carried out using transwell chambers with 6.5 mm diameter polycarbonate filters (8 μm pore size, Corning, USA) coated with 35 μL Matrigel (Becton, Dickinson and Company, USA). The MUM-2B cell suspensions(1 × 10^5^) in RPMI-1640 media were added to the upper well of transwell chambers. RPMI-1640 supplemented with 10% FBS was loaded in the lower well as a chemoattractant. Apatinib (0, 0.01, 0.05, 0.1, 0.5 μmol/L) was contained in both upper and lower wells. The inserts were incubated at 37 °C for 24 h and then, the upper Matrigel-coated surface was wiped off using a cotton swab. Cells migrating through the filters were fixed, stained with Crystal violet (Shanghai biyuntian Biological Technology Co. Ltd, China), and counted under a light microscope.

### Immunohistochemistry

Tissue samples harvested from the sacrificed mice were fixed in 10% formalin, paraffin-embedded, and sectioned. Tissue sections 5 mm in thickness were dewaxed and incubated with 0.01 M sodium citrate for antigen retrieval. The slides were rinsed in PBS and incubated overnight at 4°C with the primary antibody. Biotinylated goat anti-rabbit anti-immunoglobulin G (IgG) was used as the secondary antibody. Steps were then performed using the immunostaining kit, following the manufacturer’s instructions. The primary antibody: rabbit antimouse VEGFR-2 (Bio-World, USA), and rabbit antimouse ERK-1/2, rabbit antimouse PI3K, and rabbit antimouse MMP-2 (all from Abcam Trading Co., Ltd, Shanghai, China). The expressions of VEGFR-2, ERK-1/2, PI3K, and MMP-2 were determined by immunohistochemical staining in tumor tissue, and the cytoplasm of malignant melanoma tumor cells was all stained brown. We tested the area of stained brown protein (a), the total area (b), and the average gray level (c) per immunohistochemical stained section at 400× magnification. $\text{The}\;\text{amount}\;\text{of}\;\text{protein}\;\text{expression}=\frac{a}{b}\times 100\%\times c$ [15].

### CD31-PAS dual staining

Five micrometer paraffin sections were routinely deparaffinized and dehydrated. First, CD31 immunohistochemical staining was applied to the sections, using the immunohistochemistry method described above, with primary antibody:rabbit antimouse CD31 (1:200 Bio-World, USA). Sections were then treated with 0.5% periodic acid solution (PAS) for 10 min and rinsed with distilled water for 2–3 min. In a dark chamber, sections were treated with Schiff solution for 15–30 min. After rinses with distilled water, sections were counterstained with hematoxylin[3,8].

Ten fields of vision of the phase contrast microscope of each pathological section were selected for counting of the number of endothelial-dependent vessels and VM at 200× and 400× magnification. The average number of endothelial-dependent vessels and VM, that are quantification of the microvessel density (MVD) and the vasculogenic mimicry density (VMD) in tumors were calculated, as reported previously. All these counts were performed blindly.

### Western blotting

The tissues were homogenized in 0.5 ml Hepes (50 mM, pH 7.5) containing 100 mM NaCl, 1 mM CaCl_2_, 1 mM dithiothreitol, 1% ethylene glycol-bis(aminoethyl ether)- tetraacetic acid 1% Triton X- 100 and proteinase inhibitors. Protein extracts were kept in ice for 30 min and then centrifuged at 14,000 g at 4°C for 30 min. Protein concentrations were determined using a bicinchoninic acid protein assay reagent kit. Protein samples (20 mg) were mixed with equal volumes of loading buffer (20% glycerol, 4% sodium dodecyl sulfate, and 100 mM TrisHCl, pH 6.8) and then boiled for 5 min in the presence of b-mercaptoethanol. Proteins were separated in 8% sodium dodecyl sulfate-polyacrylamide gels at 100 V for 2 h and then electrotransferred to nitrocellulose membranes at 270 mA for 2 h. Membranes were blocked with 5% non-fat dry milk in PBS with 0.1% Tween 20 for 1 h at room temperature. Then, membranes were incubated with anti- VEGFR-2 (Bio-World, USA), anti- ERK-1/2 (Abcam Trading Co., Ltd, Shanghai, China), anti- PI_3_K (Abcam Trading Co., Ltd, Shanghai, China) and anti- MMP-2(Abcam Trading Co., Ltd, Shanghai, China) overnight at 4°C and finally with a horseradish peroxidase-conjugated anti-mouse IgG for 1 h at room temperature after washing with TBS containing 0.1% Tween 20. Proteins were visualized by enhanced chemiluminescence reagents after washing. Protein expression was semi-quantified using an image analysis system.

### Statistical analyses

Statistical analyses were carried out using SPSS 23.0 software (SPSS Inc., USA), with Student’s *t*-test for two groups, or one-way ANOVA for multiple groups. Means were considered significantly different when *p* < 0.05.

## Results

### Inhibitory effects of apatinib on vasculogenic mimicry of MUM-2B cells cultured in three-dimensional Matrigel

**Fig 1:**
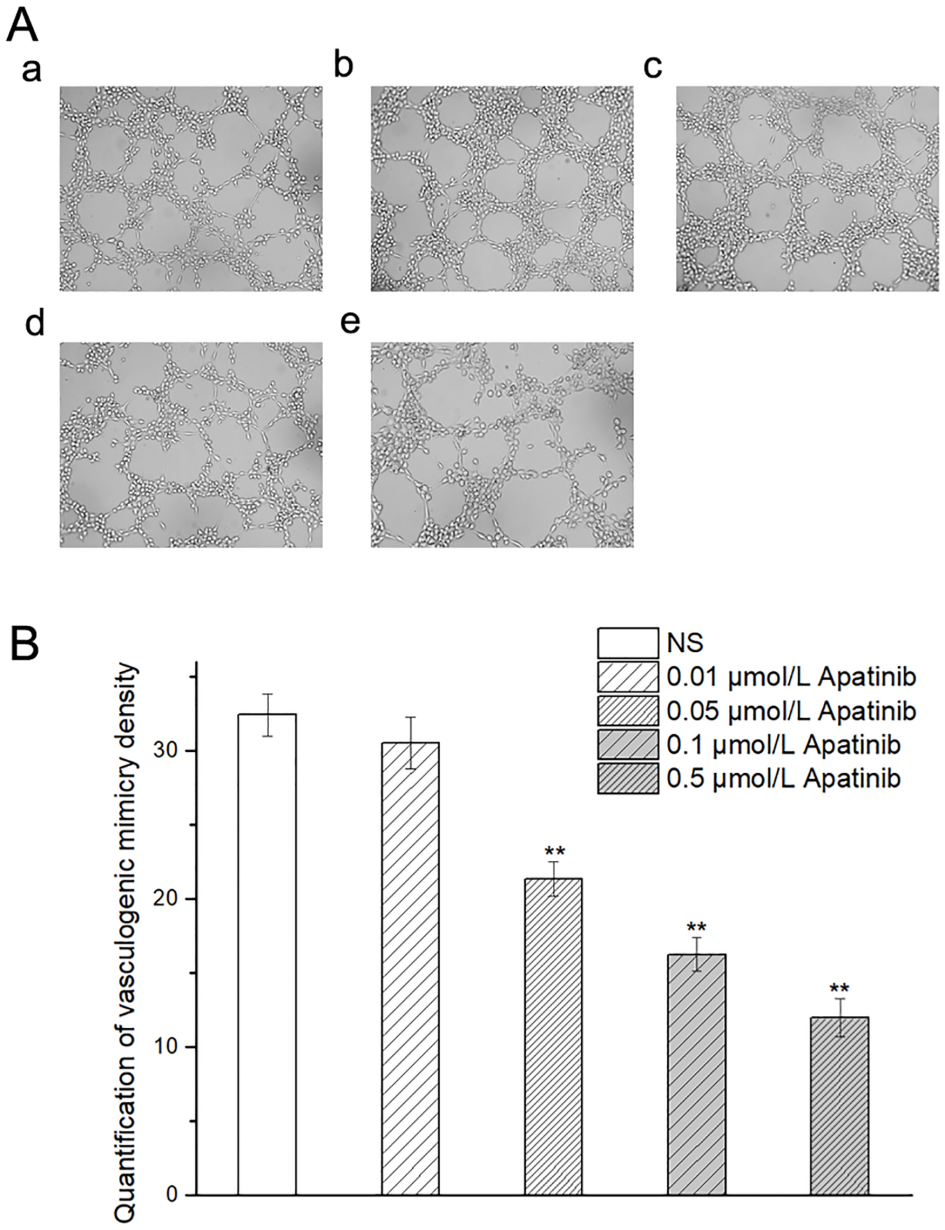
**A a-e**: the formation of VM in MUM-2B cells with different concentrations of Apatinib (0,0.01,0.05,0.1,0.5μmol/L) in the three-dimensional Matrigel at 200× magnification. **B**:The quantification of vasculogenic mimicry density. *p < 0.05 and **p < 0.01 vs.NS.

In three-dimensional Matrigel, MUM-2B cells formed VM which was directly surrounded by tumor cells, absent of endothelial cells in the inner wall of the pipe. In different concentrations of apatinib, the formation of VM in MUM-2B cells was observed under a phase contrast microscope at 200× magnification (Fig 1Aa–1Ae).

As seen in Fig 1B, the VMD in the three-dimensional culture Matrigel in 0.05 μmol/l apatinib group (21.33 ± 1.16 vs 32.40 ± 1.13, *p* < 0.01), 0.1 μmol/l apatinib group (16.24 ± 1.14 vs. 32.40 ± 1.13, *p* < 0.01), or 0.5 μmol/l apatinib group (11.95 ± 1.29 vs.32.40 ± 1.13, *p* < 0.01) was significantly lower than that in the NS group. There were no significant differences between the NS group and the 0.01 μmol/l apatinib group (30.51± 1.72 vs 32.40 ± 1.13, *p* = 0.397). Thus, we find that, *in vitro*, apatinib can reduce the number of VM in MUM-2B cells cultured in the three-dimensional culture medium at a certain dose.

### Inhibitory effects of apatinib on cell proliferation and invasion of MUM-2B cells in vitro

**Fig 2:**
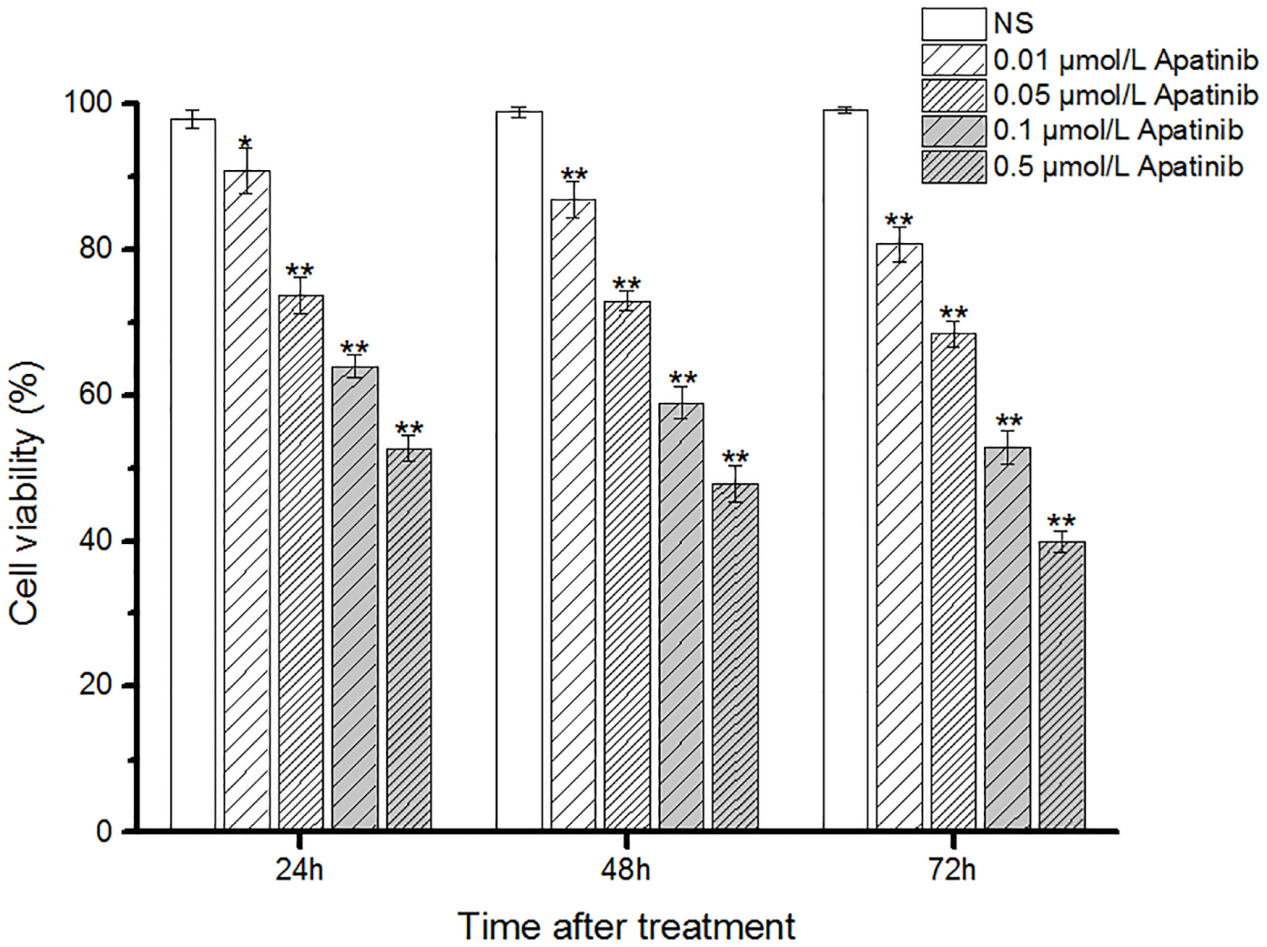
Inhibitory effects of apatinib on MUM-2B cell proliferation. Proliferation activity of MUM-2B cells was determined by the MTT assay after incubation for 24, 48, and 72h with different concentrations of Apatinib (0.01,0.05,0.1,0.5μmol/L).*p < 0.05 and **p < 0.01 vs.NS.

To examine whether apatinib had any effects on the proliferation and invasion of melanoma cells, we cultured MUM-2B cells in the presence of apatinib. Proliferation activity of MUM-2B cells was determined by the MTT assay after incubation for 24, 48, and 72 h with varying concentrations of apatinib (0.01, 0.05, 0.1, 0.5 μmol/L). As shown in Fig 2, compared with the NS group, treatment with 0.05, 0.1, 0.5 μmol/L apatinib for 24 h resulted in a significant inhibition of cell growth. After treatment with apatinib for 48 or 72 h, all doses (0.01, 0.05, 0.1, 0.5 μmol/L) showed a significant reduction of cell growth of MUM-2B cell lines.

**Fig 3:**
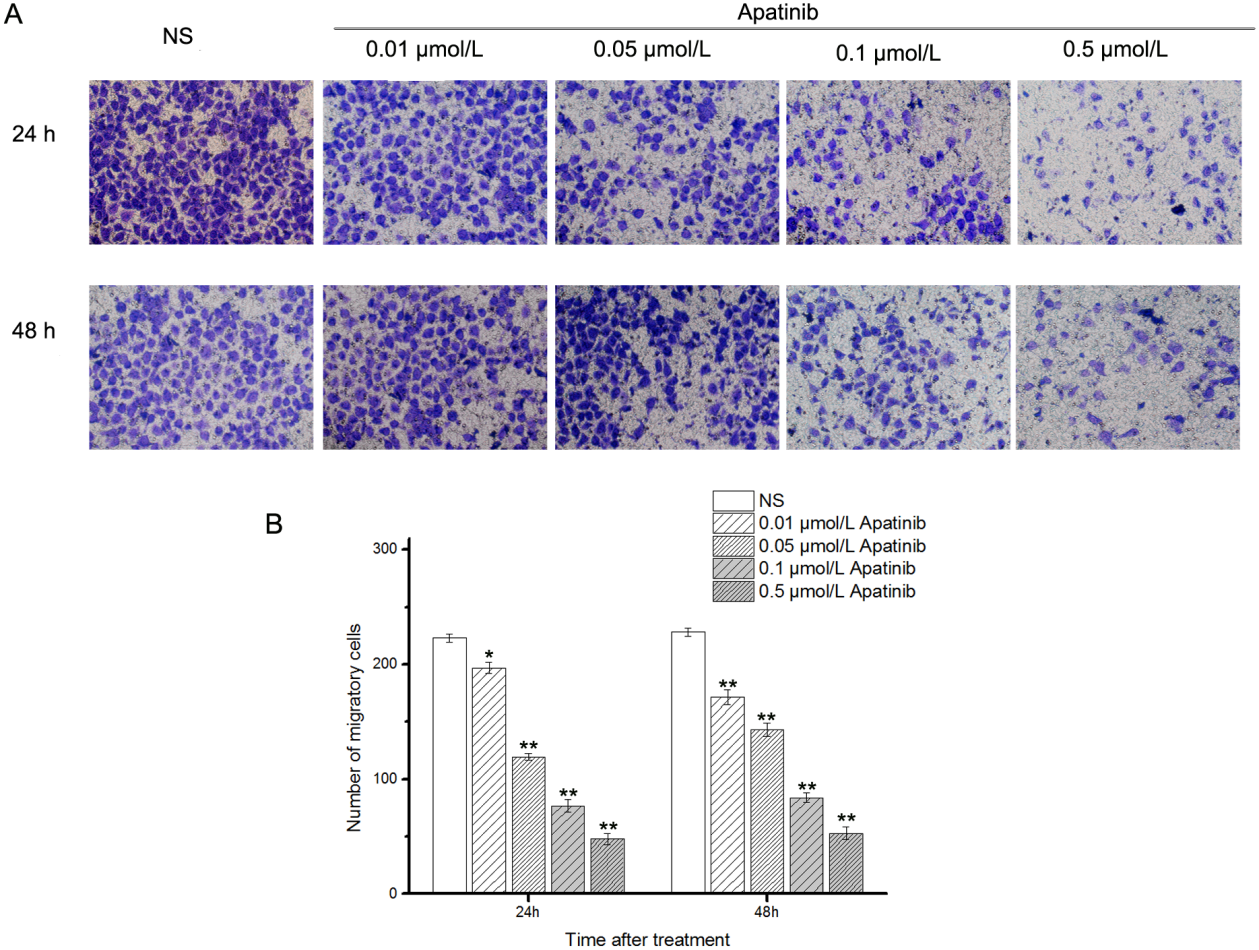
**A**:Inhibitory effects of apatinib on MUM-2B cell invasion. Invasion activity of MUM-2B cells was determined by the transwell chambers coated with Matrigel after incubation for 24 h and 48 h with different concentrations of Apatinib. **B**:The quantification of migrating cell number. *p < 0.05 and **p < 0.01 vs.NS.

Invasion activity of MUM-2B cells was determined by transwell chambers coated with Matrigel after incubation for 24 h and 48 h with different concentrations of apatinib (0.01, 0.05, 0.1, 0.5 μmol/L). As shown in Fig 3, compared with the NS group, apatinib incubation (0.01, 0.05, 0.1, 0.5 μmol/L) for 24 or 48 h all significantly decreased the number of cells migrating in a dose-dependent manner.

### *In vivo* tumor growth inhibition by apatinib

**Fig 4:**
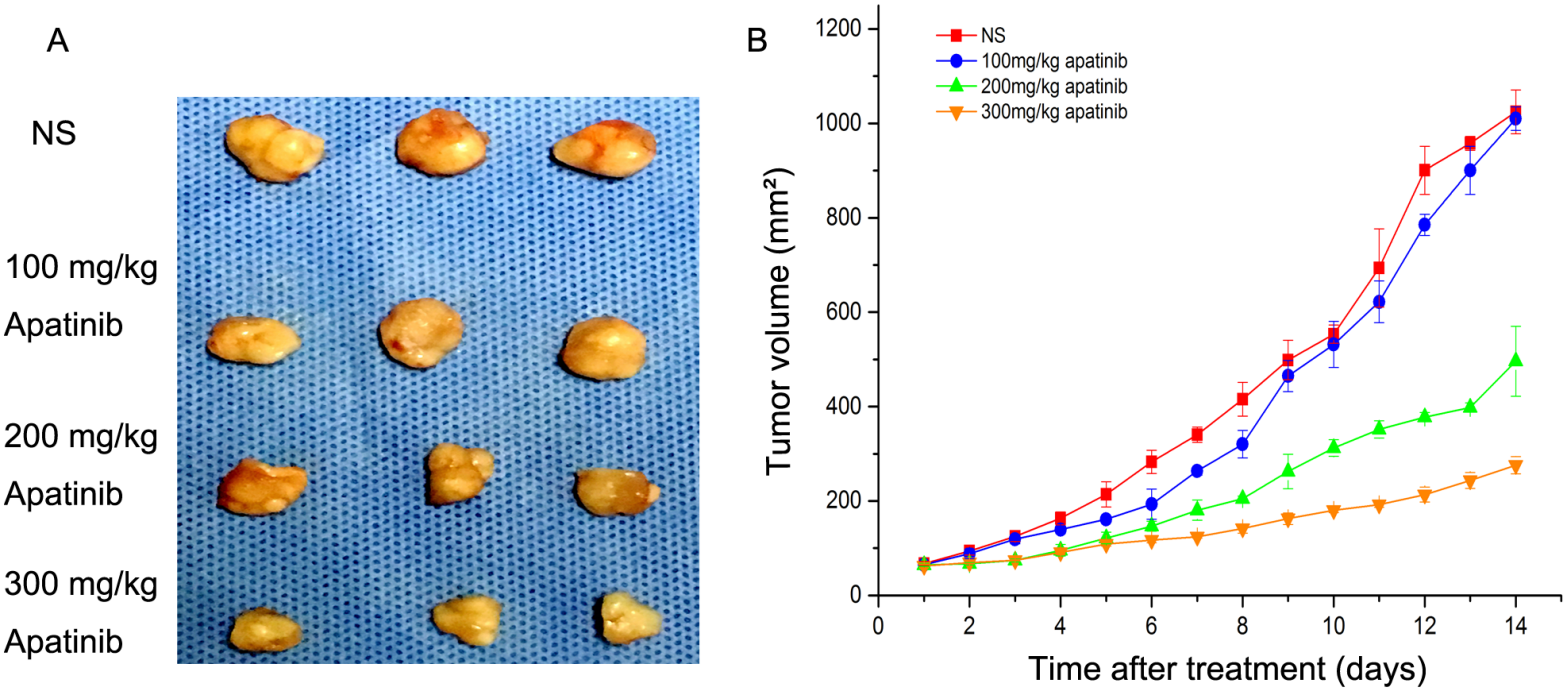
Tumor growth in subcutaneous melanoma cancer model. **A**: Suppression of subcutaneous tumor growth in each group. **B**:The final tumor volume on day 14.

To evaluate the effect of apatinib on melanoma mouse xenografts, tumor volume was measured and plotted after each treatment. The curves of the 200 mg/kg and 300 mg/kg apatinib groups were each more gentle compared to the control group (Fig 4). After 14 days, the tumor volume of the 200 mg/kg apatinib group (496.07 ±74.2 mm^3^) and 300 mg/kg apatinib group (275.8 ±18.07 mm^3^) was significantly smaller than NS group (1024.4 ± 46.2 mm^3^) (*p* < 0.01). There were no significant differences between the NS group and the 100 mg/kg apatinib group (1024.4 ± 46.2 vs. 1009.8± 24.98, *p* = 0.108).

The inhibition rate on day 14 was 1.24% in the 100 mg/kg apatinib group, 54.92% in the 200 mg/kg apatinib group, and 77.68% in the 300 mg/kg apatinib group. These results demonstrated that treatment with medium and high doses of apatinib (200 and 300 mg/kg) was the most effective treatment in reducing the tumor volume. There were no significant differences in the body weight of mice between the four groups, before and after treatment (data not shown).

### *In vivo*, apatinib inhibits angiogenesis and formation of vasculogenic mimicry in melanoma cancer xenografts

Different from the traditional endothelial-dependent vessels, vasculogenic mimicry is directly surrounded by tumor cells absent of endothelial cells in the inner wall of the pipe. CD31-PAS double staining was used to distinguish VM and endothelial-dependent vessels. CD31 is a marker of endothelial cells, and the basement membrane is positive for PAS. Therefore, we counted PAS-positive and CD31-positive as endothelial-dependent vessels and PAS-positive and CD31-negative vessels as VM.

**Fig 5:**
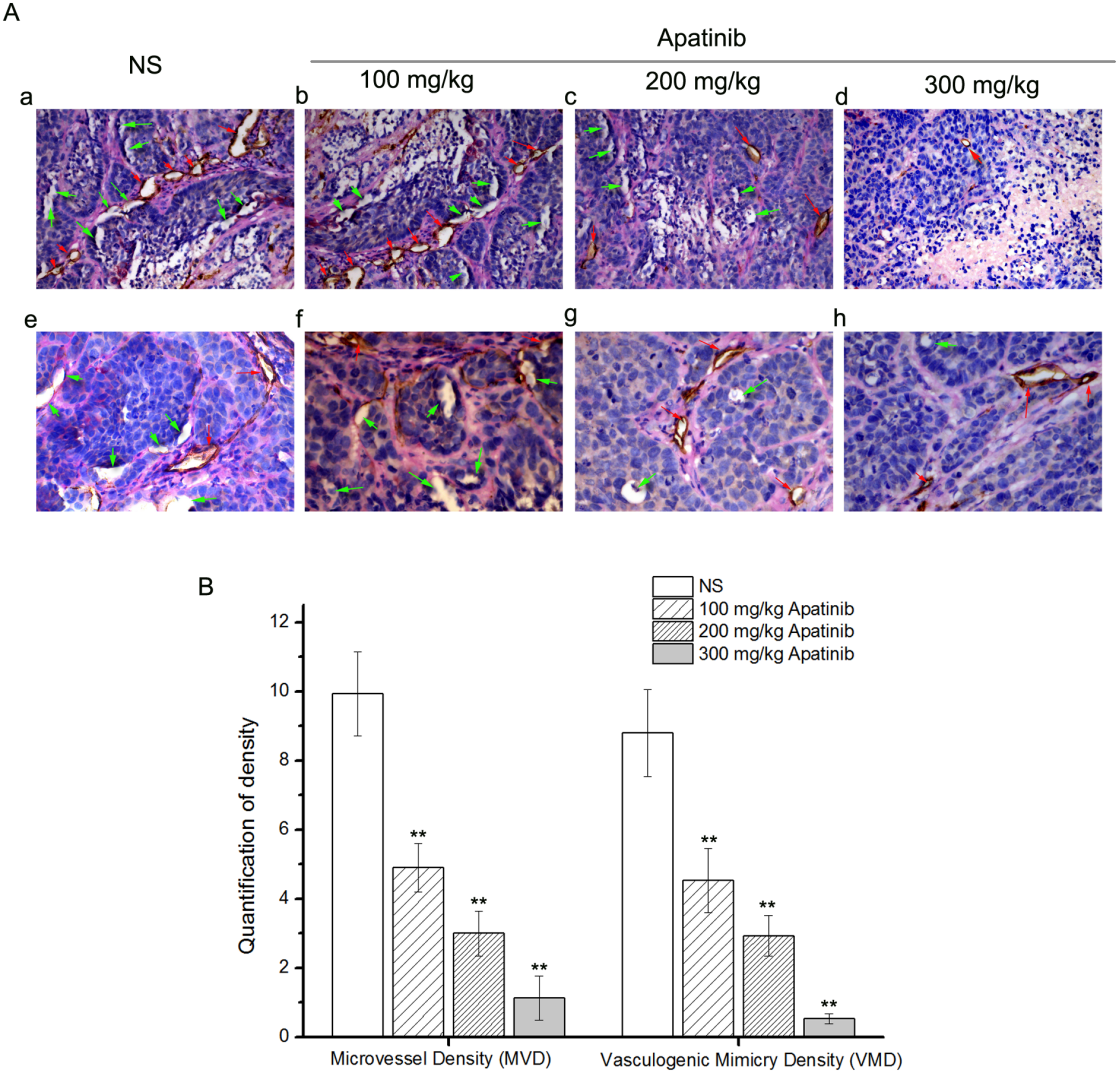
**A**:The traditional endothelial-dependent vessels and VM with CD31-PAS double staining with different concentrations of apatinib in models of melanoma cancer **(A a-d**: at 200×magnification, **A e-h**: at 400× magnification, **green *arrow***: structure of VM, **red *arrow***: structure of endothelial-dependent vessels). **B**:The quantification of microvessel density (MVD) and vasculogenic mimicry density (VMD). *p < 0.05 and **p < 0.01 vs.NS.

As shown in Fig 5, tumors in the 100 mg/kg apatinib group (4.90 ± 0.70 vs. 9.93 ± 1.22, *p* < 0.01), 200 mg/kg apatinib group (3.00 ± 0.65 vs. 9.93 ± 1.22, *p* < 0.01), 300 mg/kg apatinib group (1.13 ± 0.64 vs. 9.93 ± 1.22, *p* < 0.01) formed significantly less endothelial-dependent vessels than the NS group (9.93 ± 1.22).

As aslo shown in Fig 5, compared with the NS group (8.80 ± 1.26), tumors in the 100 mg/kg apatinib group (4.53 ± 0.92, *p* < 0.01), and the 200 mg/kg apatinib group (2.93 ± 0.59, *p* < 0.01) formed fewer VM channels. It was difficult to find the presence of VM in the 300 mg/kg apatinib group (0.53 ± 0.15, *p* <0.01). Most of the intratumor vessels seemed to be normal blood vessels, while VM structures were frequently found in the periphery of tumors (Fig 5A). Thus, our study suggests that apatinib can inhibit angiogenesis and the formation of VM in the melanoma cancer xenografts. With increased concentrations of apatinib, these inhibitory effects on angiogenesis and VM are stronger (Fig 5B).

### Downregulation of VEGFR-2, ERK-1/2, PI_3_K and MMP-2 expression by apatinib in mouse xenografts

#### Immunohistochemical staining results

**Fig 6:**
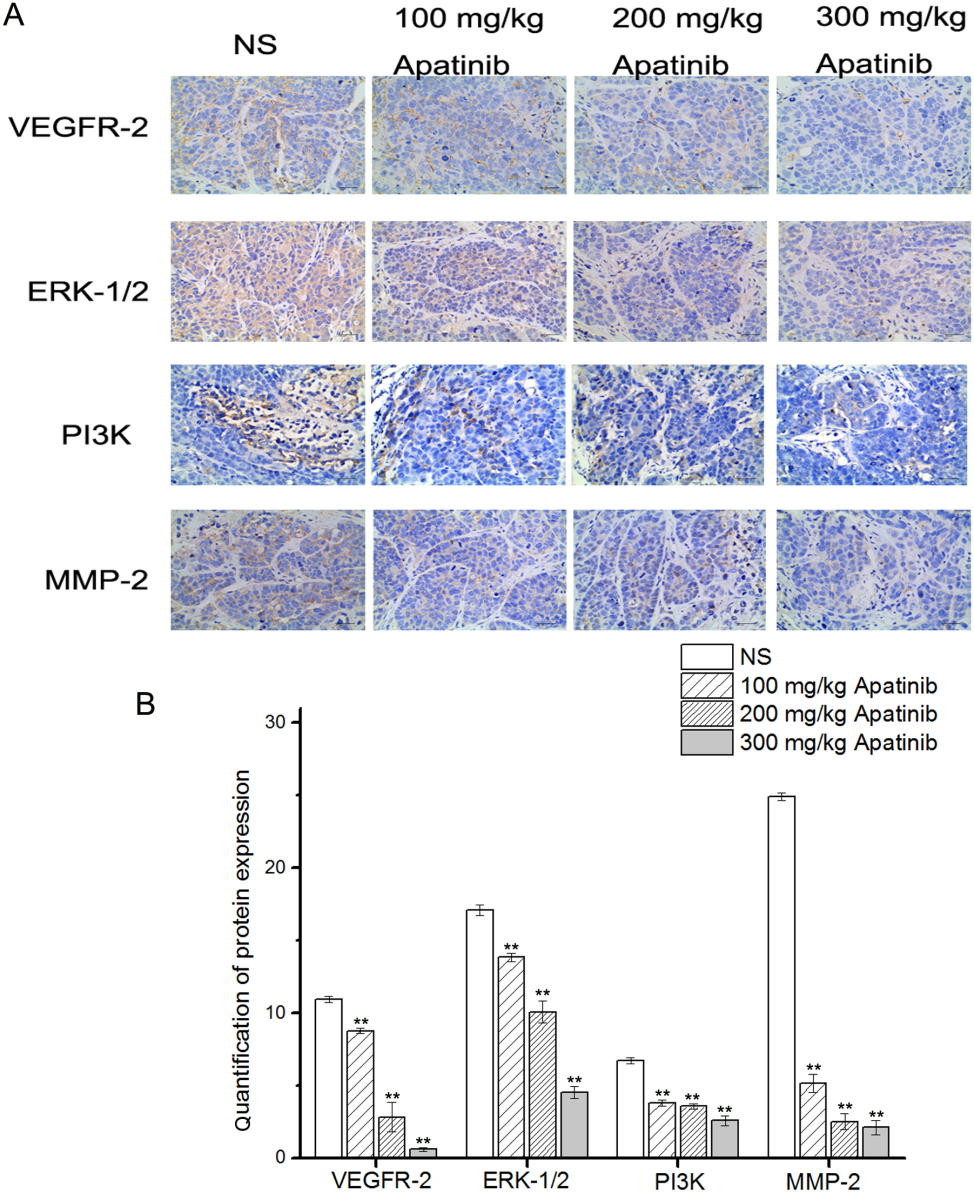
**A**: VEGFR-2, ERK-1/2, PI3K and MMP-2 immunohistochemical images of tumor tissue from mice in various groups. **B**: VEGFR-2, ERK-1/2, PI3K and MMP-2 quantitative analysis in xenografts from mice in various groups.*p < 0.05 and **p < 0.01 vs.NS. (Original magnification, 400×).

As shown in Fig 6A, the expression of VEGFR-2 in the 100 mg/kg apatinib group (8.78 ± 0.20% vs. 10.94 ± 0.22%, *p* < 0.01), 200 mg/kg apatinib group (2.83 ± 1.01% vs. 10.94 ± 0.22%, *p* < 0.01), and 300 mg/kg apatinib group (0.60 ± 0.13% vs. 10.94 ± 0.22%, *p* < 0.01) were significantly lower than that in the NS group (10.94 ± 0.22%). In addition, compared with the NS group (17.10 ± 0.38%), the expression of ERK-1/2 in the 100 mg/kg apatinib group (13.84 ± 0.3%, *p* < 0.01), 200 mg/kg apatinib group (10.07 ± 0.77%, *p* < 0.01), 300 mg/kg apatinib group (4.52 ± 0.43%, *p* < 0.01) was significantly lower.

In the 100 mg/kg apatinib group (3.81 ± 0.21%, *p* < 0.01), 200 mg/kg apatinib group (3.58 ± 0.18%, *p* < 0.01), and the 300 mg/kg apatinib group (2.59 ± 0.33%, *p* < 0.01), the expression of PI_3_K was obviously inhibited, as compared with the NS group (6.71 ± 0.22%). Moreover, the expression of MMP-2 is significantly inhibited in the 100 mg/kg apatinib group (5.15 ± 0.61%, *p* < 0.01), 200 mg/kg apatinib group (2.52 ± 0.55%, *p* < 0.01), and the 300 mg/kg apatinib group (2.12 ± 0.48%, *p* < 0.01), compared with the NS group (24.93 ± 0.26%). The quantification of determination of the positive expression ratio for VEGFR-2, ERK-1/2, PI_3_K, and MMP-2 are shown in Fig 6B.

#### Western blot results

**Fig 7:**
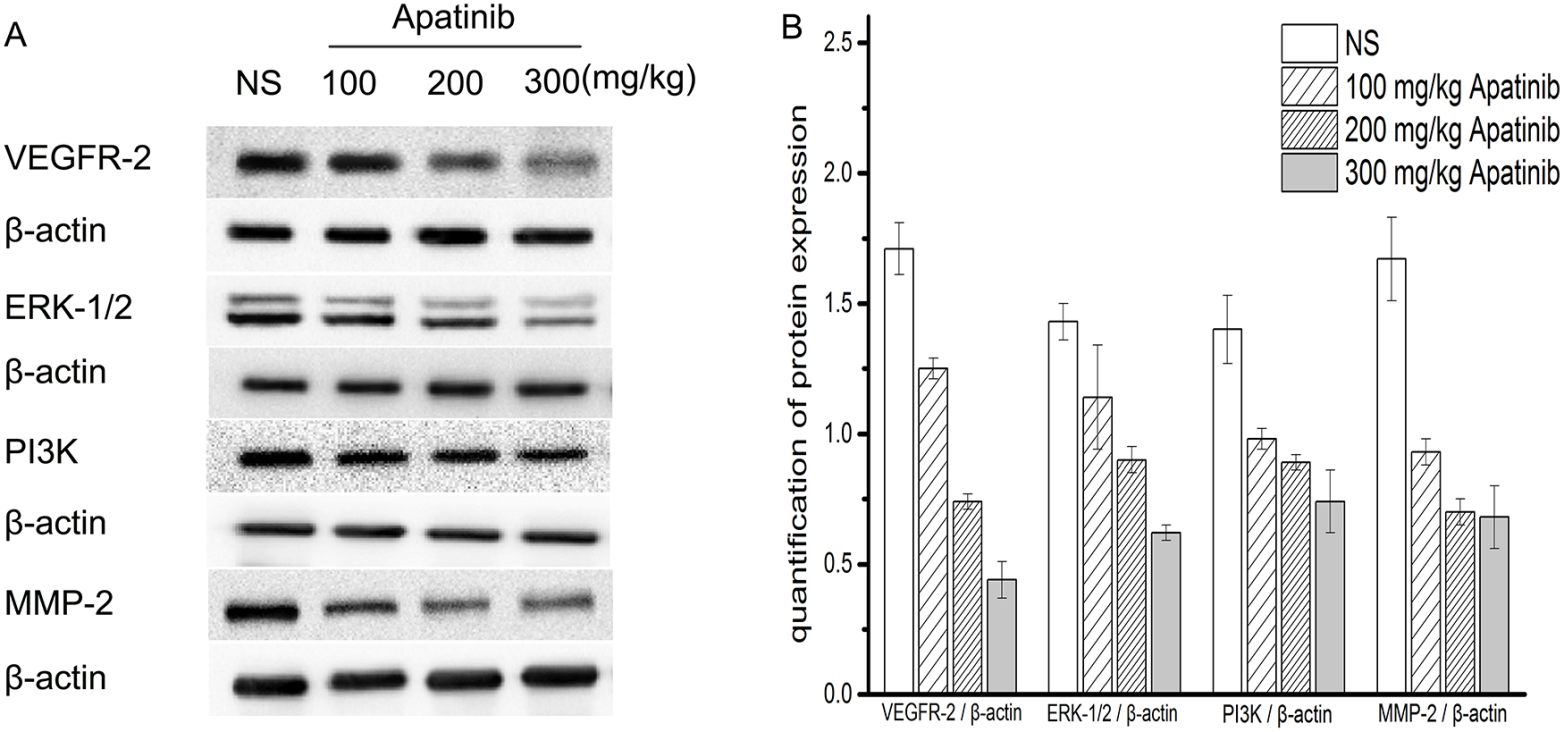
**A**:Expression of VEGFR-2,ERK-1/2,PI3K and MMP-2 in tumor tissue from mice in various groups. A representative western blot is shown. β -actin was used as a loading control. **B**:VEGFR-2/ β-actin, ERK-1/2 / β-actin, PI3K/ β-actin and MMP-2/ β-actin quantitative analysis in xenografts from mice in various groups.*p < 0.05 and **p < 0.01 vs.NS.

To further confirm our results, the expressions of VEGFR-2, ERK-1/2, PI3K and MMP-2 in mouse xenografts were analyzed by western blotting(Fig 7). Compared to the NS group (1.71 ± 0.10), mice that received 100 mg/kg apatinib (1.25 ± 0.04, *p* < 0.01), 200 mg/kg apatinib (0.74 ± 0.03, *p* < 0.01), or 300 mg/kg apatinib (0.44 ± 0.07, *p* < 0.01) therapy showed a markedly reduced level of VEGFR-2 expression. In addition, the expression of ERK-1/2 in the 100 mg/kg apatinib group (1.14 ± 0.08 vs.1.43 ± 0.07, *p* < 0.05), 200 mg/kg apatinib group (0.90 ± 0.05 vs. 1.43 ± 0.07, *p* < 0.01), or 300 mg/kg apatinib group (0.62 ± 0.03 vs. 1.43± 0.07, *p* < 0.01) were significantly lower than that in the NS group (1.43± 0.07).

Compared with the NS group (1.40 ± 0.13), the expression of PI_3_K in the 100 mg/kg apatinib group (0.98 ± 0.04, *p* < 0.01), 200 mg/kg apatinib group (0.89 ± 0.03, *p* < 0.01), 300 mg/kg apatinib group (0.74 ± 0.12, *p* < 0.01) was significantly lower. Moreover, in the 100 mg/kg apatinib group(0.93 ± 0.05, *p* < 0.01), 200 mg/kg apatinib group (0.70 ± 0.05, *p* < 0.01), and the 300 mg/kg apatinib group (0.68 ± 0.11, *p* < 0.01), MMP-2 expression was obviously inhibited, as compared with the NS group (1.67± 0.16). VEGFR-2/ β-actin, ERK-1/2 / β-actin, PI3K/ β-actin and MMP-2/ β-actin quantitative analysis in xenografts from mice in various groups are shown in Fig 7B.

In summary, our study suggests that apatinib can downregulate the expression of VEGFR-2, and inhibit the expression of ERK-1/2, PI_3_K, and MMP-2 in xenografts from mice.

## Discussion

Previous studies have explored the influence of anti-angiogenic therapies on the formation of vasculogenic mimicry (VM). For example, endostatin was reported to have no obvious inhibitory effect on the formation of VM[8]. Bevacizumab could even accelerate VM formation[3]. The effects of other angiogenesis inhibitors on VM remain unknown. In this study, we found that apatinib, a new agent for antiangiogenic therapy, restrained the formation of VM in MUM-2B cells *in vitro* and *in vivo* (Figs 1 and 5). thus providing potential targets for apatinib to treat cancer.

The tumor microcirculation plays a central role in the rapid growth of cancer cells. However, endothelium-dependent vessels are not sufficient to sustain tumor growth. Some studies have proposed that VM is an important complement of tumor microcirculation. It can provide blood supply for tumor tissue, especially at the early stage of tumor formation. The presence of VM is correlated to an increased risk of metastasis and poor clinical outcome[17–20]. Since VM vessels are formed without the contribution of endothelial cells, the angiogenesis inhibitors have shown limited effect on VM. Previous studies have demonstrated that after anti-angiogenic therapy, tumors resort to aggressive neovascularization mechanisms to cope with the therapeutic insult and thereby adopt VM as a novel neovascularization mechanism to counter the ensuing hypoxic environment within the tumor. Thus, VM was regarded as one of the mechanisms for the failure in current anti-angiogenic therapies[3,21,22]. An agent is needed to inhibit angiogenesis and VM at the same time.

Our *in vitro* study showed that MUM-2B cells had formed vascular tube-like networks in the control group (Fig 1). The number of tube-like networks were significantly lower in apatinib-treated groups compared to the control group. This clearly demonstrated the potential effect of apatinib to inhibit VM formation. In addition, the cell viability in MTT proliferation assay (Fig 2) and the number of migration cells in transwell invasion assay (Fig 3) were significantly lower in apatinib-treated groups compared to the control group. This suggested that apatinib could have inhibitory effects on cell proliferation and invasion of MUM-2B cells, which was a close relationship with the VM[17–20]. The *in vivo* study further confirmed our hypothesis. VM structures are rich in laminin, positive for PAS staining, and negative for CD31 staining. Fig 5, representing the tumor areas co-stained with PAS and CD31, shows that the apatinib-treated groups have significantly lower PAS-positive areas than the control group with a dose-dependent manner, indicting that apatinib can not only inhibit angiogenesis but also decrease the formation of VM in the melanoma cancer xenografts.

We had already known that the mechanism of apatinib is mediated by its binding to the intracellular ATP-binding site of VEGFR-2 receptor, blocking its phosphorylation and its downstream proangiogenic signaling, similar to most receptor tyrosine kinases (RTKs). VEGFR-2, as most receptor tyrosine kinases (RTKs), can induce proliferation via activation of the classical extracellular signal-regulated kinase (ERK) pathway[12–14]. It has been reported that ERK1/2 can be stimulated by VEGFR-2 receptors that induce autophosphorylation of the molecule. ERK1/2 activation stimulates its downstream signaling molecules including PI_3_K/MMP-2, which can promote the formation of VM by inducing tumor extracellular matrix (ECM) remodeling[9,23]. A number of studies have found that VEGFR-2 is not only expressed in blood and lymph vessel ECs, but is also expressed in tumor cells to mediate vasculogenesis, which may play a critical role in the formation of VM[24–26].

In this study, the expression of VEGFR-2 is downregulated after apatinib treatment, while the expression of signaling molecules including ERK1/2/PI_3_K/MMP-2 are all inhibited in the apatinib groups (Figs 6 and 7). Importantly, these signaling molecules have been implicated in the formation of VM, which are known to be coupled to the ERK pathway. Therefore, we could attribute the effect of apatinib on VM to its inhibitory effect on ERK1/2/PI_3_K/MMP-2 signaling. The exact mechanism needs further study.

In summary, our study reveals that apatinib can inhibit the development of new blood vessels and the formation of VM at the same time, which overcomes limitations of current antiangiogenic therapies. And its underlying mechanisms may provide new and potential targets for cancer therapy. Our research is the first study to display this new idea. We are confident that the advantages will bring a broader perspective of clinical application for apatinib in the treatment of tumors. In the future, we will continue to study other potential related proangiogenic signaling pathways, and find more sufficient evidence to explain why apatinib and bevacizumab have a completely different effect on VM.

## Conclusions

In conclusion, apatinib can inhibit the expression of VEGFR-2, and downregulate the ERK1/2/PI_3_K/MMP-2 signaling cascade, which may be one of the underlying mechanisms by which apatinib inhibits angiogenesis and the development of VM in models of melanoma cancer, and restrains the formation of VM by MUM-2B cells.

## Supporting information

S1 Table The quantification of the VMD in the three-dimensional Matrigel. (DOCX)

S2 Table The quantification of the MVD in tumors. (DOCX)

S3 Table The quantification of the VMD in tumors. (DOCX)

S4 Table The quantification of proliferation activity of MUM-2B cells (MTT 24h). (DOCX)

S5 Table The quantification of proliferation activity of MUM-2B cells (MTT 48h). (DOCX)

S6 Table The quantification of proliferation activity of MUM-2B cells (MTT 72h). (DOCX)

S7 Table The quantification of invasion activity of MUM-2B cells (24h). (DOCX)

S8 Table The quantification of invasion activity of MUM-2B cells (48h). (DOCX)

S9 Table The tumor volume after different treatments each day for 2 weeks. (DOCX)

S10 Table The quantification of VEGFR-2, ERK-1/2, PI3K and MMP-2 in xenografts from mice in various groups. (DOCX)

S11 Table The quantification of VEGFR-2/ β-actin, ERK-1/2 / β-actin, PI3K/ β-actin and MMP-2/ β-actin in xenografts from mice in various groups. (DOCX)
