# Supplementary material for: In vitro and in vivo apatinib inhibits vasculogenic mimicry in melanoma MUM-2B cells
Source: PLoS One. 2018 Jul 27;13(7):e0200845. doi: 10.1371/journal.pone.0200845 (PMC6063421; doi:10.1371/journal.pone.0200845)
Supplement: S1 Table — (DOCX) [file pone.0200845.s001.docx]

**S 1 Table .**

**The quantification of the VMD in the three-dimensional matrigel**

|  | **NS** | **0.01μmol/L**  **Apatinib** | **0.05μmol/L**  **Apatinib** | **0.1μmol/L**  **Apatinib** | **0.5μmol/L**  **Apatinib** |
| --- | --- | --- | --- | --- | --- |
| **n** | 15 | 15 | 15 | 15 | 15 |
| **Mean** | 32.40**^cde^** | 30.51**^cde^** | 21.33**^abde^** | 16.24^abce^ | 11.95**^abcd^** |
| **SD** | 1.13 | 1.72 | 1.16 | 1.14 | 1.29 |

**Tips:**

**a：comparied with NS group, P<0.05 ;**

**b：comparied with 0.01μmol/L Apatinib group, P<0.05;**

**c：comparied with 0.05μmol/L Apatinib group, P<0.05;**

**d：comparied with 0.1μmol/L Apatinib group, P<0.05;**

**e：comparied with 0.5μmol/L Apatinib group, P<0.05.**
