# Supplementary material for: In vitro and in vivo apatinib inhibits vasculogenic mimicry in melanoma MUM-2B cells
Source: PLoS One. 2018 Jul 27;13(7):e0200845. doi: 10.1371/journal.pone.0200845 (PMC6063421; doi:10.1371/journal.pone.0200845)
Supplement: S2 Table — (DOCX) [file pone.0200845.s002.docx]

**S 2 Table. The quantification of the MVD in tumors**

|  | **NS** | **100mg/kg Apatinib** | **200mg/kg Apatinib** | **300mg/kg Apatinib** |
| --- | --- | --- | --- | --- |
| **n** | 15 | 15 | 15 | 15 |
| **Mean** | 9.93^bcd^ | 4.90^acd^ | 3.00^abd^ | 1.13^abc^ |
| **SD** | 1.22 | 0.70 | 0.65 | 0.64 |
