# Supplementary material for: In vitro and in vivo apatinib inhibits vasculogenic mimicry in melanoma MUM-2B cells
Source: PLoS One. 2018 Jul 27;13(7):e0200845. doi: 10.1371/journal.pone.0200845 (PMC6063421; doi:10.1371/journal.pone.0200845)
Supplement: S3 Table — (DOCX) [file pone.0200845.s003.docx]

**S 3 Table. The quantification of the VMD in tumors**

|  | **NS** | **100mg/kg Apatinib** | **200mg/kg Apatinib** | **300mg/kg Apatinib** |
| --- | --- | --- | --- | --- |
| **n** | 15 | 15 | 15 | 15 |
| **Mean** | 8.80^bcd^ | 4.53^acd^ | 2.93^abd^ | 0.53^abc^ |
| **SD** | 1.26 | 0.92 | 0.59 | 0.15 |

**Tips:**

**a：comparied with NS group, P<0.05 ;**

**b：comparied with 100mg/kg Apatinib group, P<0.05;**

**c：comparied with 200mg/kg Apatinib group, P<0.05;**

**d：comparied with 300mg/kg Apatinib group, P<0.05;**
