# Supplementary material for: In vitro and in vivo apatinib inhibits vasculogenic mimicry in melanoma MUM-2B cells
Source: PLoS One. 2018 Jul 27;13(7):e0200845. doi: 10.1371/journal.pone.0200845 (PMC6063421; doi:10.1371/journal.pone.0200845)
Supplement: S4 Table — (DOCX) [file pone.0200845.s004.docx]

**S 4 Table .**

**The quantification of proliferation activity of MUM-2B cells (MTT 24h)**

|  | **NS** | **0.01μmol/L**  **Apatinib** | **0.05μmol/L**  **Apatinib** | **0.1μmol/L**  **Apatinib** | **0.5μmol/L**  **Apatinib** |
| --- | --- | --- | --- | --- | --- |
| **Mean** | 98.8%^bcde^ | 86.7%^acde^ | 72.9%^abde^ | 58.9%^abce^ | 47.9%^abcd^ |
| **SD** | 0.7 | 2.5 | 1.3 | 2.2 | 2.5 |
