# Supplementary material for: In vitro and in vivo apatinib inhibits vasculogenic mimicry in melanoma MUM-2B cells
Source: PLoS One. 2018 Jul 27;13(7):e0200845. doi: 10.1371/journal.pone.0200845 (PMC6063421; doi:10.1371/journal.pone.0200845)
Supplement: S6 Table — (DOCX) [file pone.0200845.s006.docx]

**S 6 Table .**

**The quantification of proliferation activity of MUM-2B cells (MTT 72h)**

|  | **NS** | **0.01μmol/L**  **Apatinib** | **0.05μmol/L**  **Apatinib** | **0.1μmol/L**  **Apatinib** | **0.5μmol/L**  **Apatinib** |
| --- | --- | --- | --- | --- | --- |
| **Mean** | 99.4%^bcde^ | 80.7%^acde^ | 68.4%^abde^ | 52.9%^abce^ | 39.9%^abcd^ |
| **SD** | 0.19 | 2.4 | 1.8 | 2.3 | 1.5 |

**Tips:**

**a：comparied with NS group, P<0.05 ;**

**b：comparied with 0.01μmol/L Apatinib group, P<0.05;**

**c：comparied with 0.05μmol/L Apatinib group, P<0.05;**

**d：comparied with 0.1μmol/L Apatinib group, P<0.05;**

**e：comparied with 0.5μmol/L Apatinib group, P<0.05.**
