# Supplementary material for: In vitro and in vivo apatinib inhibits vasculogenic mimicry in melanoma MUM-2B cells
Source: PLoS One. 2018 Jul 27;13(7):e0200845. doi: 10.1371/journal.pone.0200845 (PMC6063421; doi:10.1371/journal.pone.0200845)
Supplement: S7 Table — (DOCX) [file pone.0200845.s007.docx]

**S 7 Table . The quantification of invasion activity of MUM-2B cells (24h)**

|  | **NS** | **0.01μmol/L**  **Apatinib** | **0.05μmol/L**  **Apatinib** | **0.1μmol/L**  **Apatinib** | **0.5μmol/L**  **Apatinib** |
| --- | --- | --- | --- | --- | --- |
| **Mean** | 223^bcde^ | 197^acde^ | 119.4^abde^ | 76.8^abce^ | 48^bcde^ |
| **SD** | 3.46 | 5.12 | 2.81 | 5.41 | 4.82 |
