# Supplementary material for: In vitro and in vivo apatinib inhibits vasculogenic mimicry in melanoma MUM-2B cells
Source: PLoS One. 2018 Jul 27;13(7):e0200845. doi: 10.1371/journal.pone.0200845 (PMC6063421; doi:10.1371/journal.pone.0200845)
Supplement: S8 Table — (DOCX) [file pone.0200845.s008.docx]

**S 8 Table .**

**The quantification of invasion activity of MUM-2B cells (48h)**

|  | **NS** | **0.01μmol/L**  **Apatinib** | **0.05μmol/L**  **Apatinib** | **0.1μmol/L**  **Apatinib** | **0.5μmol/L**  **Apatinib** |
| --- | --- | --- | --- | --- | --- |
| **Mean** | 228^bcde^ | 171.5^acde^ | 143.2^abde^ | 83.8^abce^ | 52.8^bcde^ |
| **SD** | 3.7 | 6.26 | 5.87 | 4.18 | 5.4 |

**Tips:**

**a：comparied with NS group, P<0.05 ;**

**b：comparied with 0.01μmol/L Apatinib group, P<0.05;**

**c：comparied with 0.05μmol/L Apatinib group, P<0.05;**

**d：comparied with 0.1μmol/L Apatinib group, P<0.05;**

**e：comparied with 0.5μmol/L Apatinib group, P<0.05.**
