# Supplementary material for: In vitro and in vivo apatinib inhibits vasculogenic mimicry in melanoma MUM-2B cells
Source: PLoS One. 2018 Jul 27;13(7):e0200845. doi: 10.1371/journal.pone.0200845 (PMC6063421; doi:10.1371/journal.pone.0200845)
Supplement: S9 Table — (DOCX) [file pone.0200845.s009.docx]

**S 9 Table .**

**The tumor volume after different treatments each day for 2 weeks**

|  | **NS** | | **The 100 mg/kg Apatinib** | | **The 200 mg/kg Apatinib** | | **The 300 mg/kg Apatinib** | |
| --- | --- | --- | --- | --- | --- | --- | --- | --- |
|  | **mean** | **SD** | **mean** | **SD** | **mean** | **SD** | **mean** | **SD** |
| **Day 1** | 67.55 | 4.8 | 64.85 | 3.23 | 64.7 | 3.01 | 62.24 | 4.36 |
| **Day 2** | 93.65 | 8.42 | 88.15 | 10.12 | 67.05 | 2.72 | 69.35 | 1.79 |
| **Day 3** | 125 | 10.69 | 119 | 9.56 | 74.05 | 3.03 | 74 | 1.37 |
| **Day 4** | 163.2 | 13.8 | 139.3 | 7.81 | 95.68 | 12.38 | 91.38 | 4.55 |
| **Day 5** | 214.1 | 26.59 | 161.2 | 7.16 | 121.6 | 12.20 | 108.6 | 3.97 |
| **Day 6** | 282.75 | 24.83 | 193 | 31.94 | 146.74 | 10.72 | 117.15 | 5.57 |
| **Day 7** | 340.2 | 16.25 | 263.7 | 8.69 | 180.65 | 21.09 | 124.35 | 3.29 |
| **Day 8** | 415.5 | 35.69 | 320.4 | 29.45 | 205.08 | 5.34 | 141.69 | 9.62 |
| **Day 9** | 498.7 | 41.54 | 464.82 | 32.96 | 262.46 | 36.72 | 162.48 | 12.37 |
| **Day 10** | 553.5 | 19.09 | 531.74 | 48.69 | 312.66 | 17.55 | 180.58 | 5.34 |
| **Day 11** | 693.4 | 82.87 | 621.98 | 44.49 | 351.72 | 18.28 | 192.36 | 5.44 |
| **Day 12** | 900.65 | 50.92 | 785 | 22.36 | 377.78 | 9.81 | 213.4 | 15.88 |
| **Day 13** | 957.53 | 14.81 | 900.65 | 50.92 | 397.96 | 9.81 | 243.6 | 16.98 |
| **Day 14** | 1024.4 | 46.20 | 1009.8 | 24.98 | 496.07 | 74.2 | 275.8 | 18.07 |
| **The inhibition rate on day 14** |  | | 1.24% | | 54.92% | | 77.68% | |
