# Supplementary material for: In vitro and in vivo apatinib inhibits vasculogenic mimicry in melanoma MUM-2B cells
Source: PLoS One. 2018 Jul 27;13(7):e0200845. doi: 10.1371/journal.pone.0200845 (PMC6063421; doi:10.1371/journal.pone.0200845)
Supplement: S10 Table — (DOCX) [file pone.0200845.s010.docx]

**S 10 Table .**

**The quantification of VEGFR-2, ERK-1/2, PI3K and MMP-2 in xenografts from mice in various groups**

|  | **n** | **VEGFR-2** | **ERK-1/2** | **PI_3_K** | **MMP-2** |
| --- | --- | --- | --- | --- | --- |
| **NS** | 10 | 10.94±0.22**^bcd^** | 17.10±0.38**^bcd^** | 6.71±0.22**^bcd^** | 24.93±0.26**^bcd^** |
| **100mg/kg Apatinib** | 10 | 8.78±0.20**^acd^** | 13.84±0.30**^acd^** | 3.81±0.21**^ad^** | 5.15±0.61**^acd^** |
| **200mg/kg Apatinib** | 10 | 2.83±1.01**^abd^** | 10.07±0.77**^abd^** | 3.58±0.18**^ad^** | 2.52±0.55**^ab^** |
| **300mg/kg Apatinib** | 10 | 0.60±0.13**^abc^** | 4.52±0.43**^abc^** | 2.59±0.33**^abc^** | 2.12±0.48**^ab^** |

**Tips:**

**a：comparied with NS group, P<0.05 ;**

**b：comparied with 100mg/kg Apatinib group, P<0.05;**

**c：comparied with 200mg/kg Apatinib group, P<0.05;**

**d：comparied with 300mg/kgApatinib group, P<0.05;**
