# Supplementary material for: In vitro and in vivo apatinib inhibits vasculogenic mimicry in melanoma MUM-2B cells
Source: PLoS One. 2018 Jul 27;13(7):e0200845. doi: 10.1371/journal.pone.0200845 (PMC6063421; doi:10.1371/journal.pone.0200845)
Supplement: S11 Table — (DOCX) [file pone.0200845.s011.docx]

**S 11 Table .**

**The quantification of VEGFR-2/ β-actin, ERK-1/2 / β-actin, PI3K/ β-actin and MMP-2/ β-actin in xenografts from mice in various groups**

|  | **n** | **VEGFR-2/ β-actin** | **ERK-1/2/ β-actin** | **PI_3_K/ β-actin** | **MMP-2/ β-actin** |
| --- | --- | --- | --- | --- | --- |
| **NS** | 10 | 1.71±0.1^bcd^ | 1.43±0.07^bcd^ | 1.4±0.13^bcd^ | 1.67±0.16^bcd^ |
| **100mg/kg Apatinib** | 10 | 1.25±0.04^acd^ | 1.14±0.2^acd^ | 0.98±0.04^acd^ | 0.93±0.05^acd^ |
| **200mg/kg Apatinib** | 10 | 0.74±0.03^abd^ | 0.9±0.05^abd^ | 0.89±0.03^abd^ | 0.7±0.05^abd^ |
| **300mg/kg Apatinib** | 10 | 0.44±0.07^abc^ | 0.62±0.03^abc^ | 0.74±0.12^abc^ | 0.68±0.12^abc^ |

**Tips:**

**a：comparied with NS group, P<0.05 ;**

**b：comparied with 100mg/kg Apatinib group, P<0.05;**

**c：comparied with 200mg/kg Apatinib group, P<0.05;**

**d：comparied with 300mg/kgApatinib group, P<0.05;**
